# T_FH_2 cells associate with enhanced humoral immunity to SARS‐CoV‐2 inactivated vaccine in patients with allergic rhinitis

**DOI:** 10.1002/ctm2.717

**Published:** 2022-01-26

**Authors:** Yin Yao, Zhe‐Zheng Wang, Ao Huang, Yan Liu, Nan Wang, Zhi‐Chao Wang, Lin Yang, Hui‐Jun Li, Jun‐Gang Xie, Rong‐Fei Zhu, Li‐Ming Cheng, Di Yu, Zheng Liu

**Affiliations:** ^1^ Department of Otolaryngology‐Head and Neck Surgery, Tongji Hospital, Tongji Medical College Huazhong University of Science and Technology Wuhan China; ^2^ Department of Allergy, Tongji Hospital, Tongji Medical College Huazhong University of Science and Technology Wuhan China; ^3^ Department of Laboratory Medicine, Tongji Hospital, Tongji Medical College Huazhong University of Science and Technology Wuhan China; ^4^ Department of Respiratory and Critical Care Medicine, Tongji Hospital, Tongji Medical College Huazhong University of Science and Technology Wuhan China; ^5^ The University of Queensland Diamantina Institute, Faculty of Medicine The University of Queensland Brisbane Queensland Australia

To the Editor:

We report that allergic rhinitis (AR) patients displayed enhanced humoral immune response to SARS‐CoV‐2 inactivated vaccine as compared to healthy controls, and highlight a pivotal role of type 2 T follicular helper cells in this response. Vaccination is considered as the best strategy to reduce SARS‐CoV‐2 infections and prevent severe illness after breakthrough infections.^1^, ^2^ Although SARS‐CoV‐2 vaccines are highly protective against COVID‐19 in healthy individuals, the vaccine efficacy may be compromised in patients with pre‐existing conditions, resulting in dysregulated immune systems. Recent data described poorer humoral immunity to COVID‐19 vaccines in individuals with kidney transplantation, undertaking dialysis, or cancer, compared to healthy controls.^1^, ^2^ Allergic diseases, including AR, atopic dermatitis and asthma, are caused by the immune system hypersensitivity to innocuous environmental antigens and characterized by skewed type 2 immune responses.^3^ Such chronic immunological disorders are highly prevalent and estimated to affect up to 50% of the world population.^3^ Allergic airway diseases have been revealed to have a potential protective role in COVID‐19, possibly because of the reduction of SARS‐CoV‐2 receptor angiotensin‐converting enzyme 2 in airway epithelial cells caused by type 2 cytokines and the abundant infiltration of eosinophils in airways.^4^ However, there are no studies formally investigating whether allergic diseases modulate the humoral response following vaccination against SARS‐CoV‐2. In the present study, we compare immunological response after two‐dose inactivated SARS‐CoV‐2 immunization between healthy subjects and patients with AR.

We conducted a prospective study to evaluate the potential impact of AR on the immune response after two‐dose inactivated SARS‐CoV‐2 immunization (Figure 1A). The trial was registered at https://clinicaltrials.gov (NCT05009134). Twenty‐five healthy adults and 32 patients with AR were recruited at Tongji Hospital (Table 1). AR was diagnosed based on the concordance between typical allergic symptoms and atopic status.^5^ Atopic status was assessed by skin‐prick testing (SPT) and/or allergen‐specific immunoglobulin E (IgE) levels.^6^ AR patients had at least 1‐year disease history and had never been infected with SARS‐CoV‐2. The exclusion criteria included (i) the presence of sinusitis, (ii) pregnancy or breastfeeding, (iii) with cardiovascular diseases, severe immunologic diseases, chronic obstructive pulmonary disease, chronic infections, diabetes, tumours, chronic kidney diseases or stroke, and (iv) use of intranasal steroid or antihistamines in the previous 1 week or oral steroids in the previous 3 months before this study. Healthy subjects had negative SPT and specific IgE test and had no history of allergy.^6^ All participants received two doses of inactivated SARS‐CoV‐2 vaccines (WIBP‐CorV, Sinopharm, Wuhan) with 1 month apart. Peripheral blood samples were collected on days 0, 7, 30 (before the second dose), 37 (7 days post the second dose) and 60 (30 days post the second dose) to analyze humoral immune responses to vaccination (Figure 1A). None was lost to follow‐up. In addition, a complementary approach was adopted to further investigate the influence of AR on the antibody response to natural SARS‐CoV‐2 infection in 78 patients recovered from COVID‐19 (64 control subjects without AR and 14 AR patients), whose peripheral bloods were collected at 10–12 months post the infection. The Ethics Committee of Tongji Hospital reviewed and approved this trial, and informed consent was provided by every participant. More information regarding subjects and methods is provided in Tables S1–S3.

**FIGURE 1:**
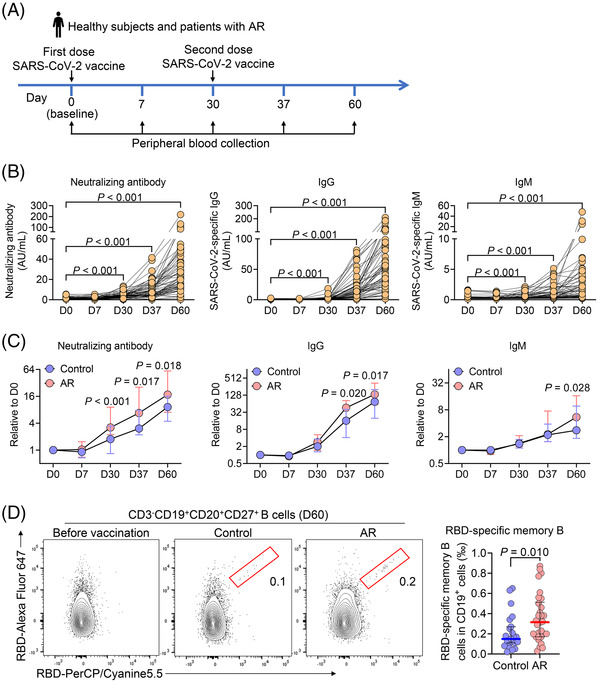
Enhanced antibody responses in patients with allergic rhinitis (AR) following inactivated SARS‐CoV‐2 vaccination. (A) Healthy subjects (*n* = 25) and patients with AR (*n* = 32) were enrolled and received inactivated SARS‐CoV‐2 vaccine at days 0 and 30. Peripheral blood was collected at days 0 (baseline), 7, 30, 37 and 60. (B) Plasma neutralizing antibodies against the RBD of the SARS‐CoV‐2 S1 protein and IgG and IgM against the SARS‐CoV‐2 S and N proteins were measured by chemiluminescent immunoassay. Data are analyzed by two‐sided paired‐sample *t*‐test. (C) Fold changes of SARS‐CoV‐2 neutralizing antibody, IgG and IgM in control subjects and AR patients by normalizing to the baseline levels. (D) Frequencies of circulating RBD‐specific memory B cells at day 60 were analyzed by flow cytometry. Numbers indicate the frequency of cells in the gated region. For (C) and (D), data are presented as median and interquartile range and analyzed by Mann–Whitney *U*‐test

**TABLE 1:** Demographic characteristics of subjects in the study of inactivated SARS‐CoV‐2 vaccination

	**Healthy subjects**	**AR patients**	***p*‐Value**
Total subjects, *n*	25	32	–
Gender, male/female	9/16	11/21	.899
Age (years)	24 (23, 27)	24 (22, 26)	.197
Body mass index	21.1 (19.1, 22.3)	20.8 (19.2, 22.4)	.908
Patients with asthma, *n* (%)	0	1 (3.1%)	1.000
Patients with atopic eczema, *n* (%)	0	1 (3.1%)	1.000
Sensitization pattern, *n* (%)			
Dermatophagoides pteronyssinus	0	30 (94.8%)	
Dermatophagoides farinae	0	30 (94.8%)	
Cockroach	0	2 (6.4%)	
Cat dander	0	5 (15.6%)	
Dog dander	0	4 (12.5%)	
Artemisia	0	1 (3.1%)	
Platanus	0	1 (3.1%)	
Alternaria	0	3 (9.4%)	
VAS symptom score	0 (0, 0)	5.0 (3.3, 7.0)	<.001

*Note*: For continuous variables, data are expressed as medians and interquartile ranges.

Abbreviations: AR, allergic rhinitis; VAS, visual analogue scale.

There was no noticeable difference in the incidence of adverse reactions between healthy subjects and patients with AR (Table S1). Protective antibody responses to WIBP‐CorV were assessed by the detection of neutralizing antibodies targeting the receptor‐binding domain (RBD) of SARS‐CoV‐2 spike (S) 1 protein and IgG and IgM against the S and nucleocapsid proteins. The inactivated SARS‐CoV‐2 vaccine elicited robust serological responses, with gradual increase in neutralizing antibody, IgG and IgM following vaccination (Figure 1B). Although neutralizing antibody, IgG and IgM levels were low following the first immunization, the second injection boosted their titres by ∼35‐, 161‐ and 16‐fold (mean) at day 60 relative to day 0 (baseline), respectively (Figure S1A). As reported in COVID‐19 infection or vaccination,^7^ the levels of neutralizing antibody strongly correlated with IgG levels (Figure S1B).

Of a particular interest, serological responses in AR patients were stronger than those in healthy controls, showing higher levels of neutralizing antibody at days 30, 37 and 60, and anit‐SARS‐CoV‐2 IgG on days 37 and 60, and anit‐SARS‐CoV‐2 IgM on day 60 (Figure 1C; Figure S1C). Higher frequencies of CD19^+^CD27^+^CD38^++^ circulating plasmablasts were found in patients with AR at day 60, although there was no noticeable difference in CD3^–^CD19^+^ total B cells and its subsets (IgD^–^CD27^–^ double negative, IgD^–^CD27^+^ switched memory, IgD^+^CD27^–^ naive and IgD^+^CD27^+^ nonswitched memory B cells) between healthy subjects and AR patients (Figures S2 and S3). To explore the immune memory after vaccination, we also analyzed memory B‐cell response specific to RBD in the peripheral blood (Figure S2). Flow cytometric analysis revealed higher frequencies of RBD‐specific memory B cells, again, in AR patients compared to healthy subjects at day 60 (Figure 1D). As the negative controls, RBD‐specific memory B cells were undetectable in samples collected before vaccination (Figure 1D). These results suggest AR patients demonstrated enhanced immune responses to inactive SARS‐CoV‐2 vaccine than control subjects.

T follicular helper (T_FH_) cells essentially support B cells for the generation of neutralizing antibodies and long‐lived humoral immunity after vaccination.^8^ We assessed vaccination‐induced T_FH_ activation by examining circulating CXCR5^+^ICOS^high^PD‐1^high^ T_FH_ (ICOS^high^PD‐1^high^ cT_FH_) cells (Figure S4).^6^ In both groups, inactivated SARS‐CoV‐2 vaccination induced a modest expansion of ICOS^high^PD‐1^high^ cT_FH_ cells, 7 days after the first immunization and a drastic expansion 7 days after the second immunization (day 37), while there was no significant difference in the frequencies of ICOS^high^PD‐1^high^ cT_FH_ cells at all the time points between the two groups (Figure 2A). To reduce the impact introduced by individual variation, we normalized the frequencies of ICOS^high^PD‐1^high^ cT_FH_ cells to the baseline at day 0 and detected higher frequencies of ICOS^high^PD‐1^high^ cT_FH_ cells at days 7, 37 and 60 in AR patients than control subjects (Figure 2B). In contrast, total CD4^+^ T cells, CXCR5^+^ total cT_FH_ cells, T_REG_ cells and T_FR_ cells were not affected by vaccination and remained comparable between two groups (Figure S5A,B). Therefore, both AR patients and control subjects mounted potent T_FH_ activation by two‐dose of WIBP‐CorV vaccination, with a stronger signature by the former group.

**FIGURE 2:**
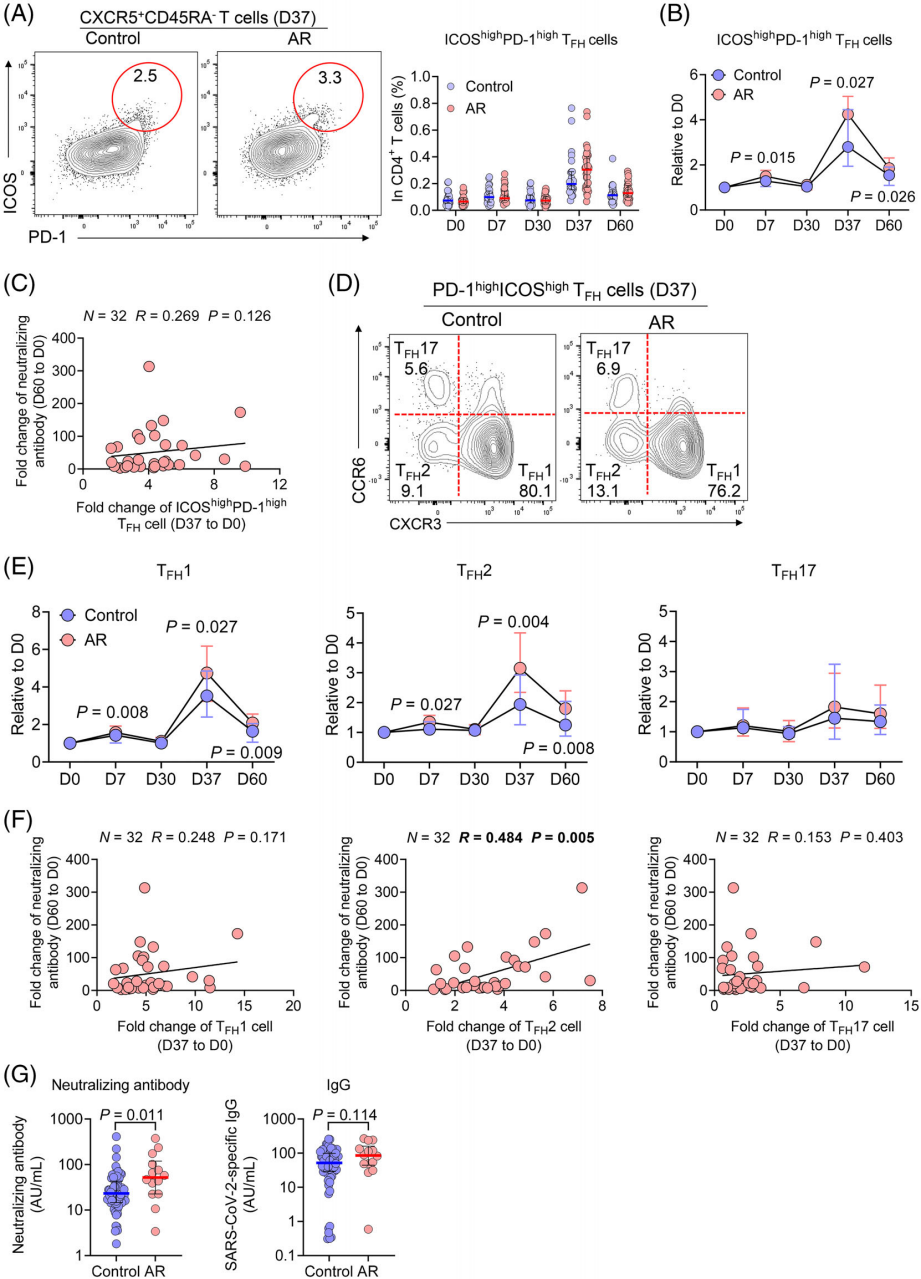
Increased circulating T_FH_2 cells associate with protective antibodies in patients with allergic rhinitis (AR) following vaccination. (A) Frequencies of circulating ICOS^high^PD‐1^high^ T_FH_ cells were analyzed by flow cytometry. (B) Changes of ICOS^high^PD‐1^high^ T_FH_ cells at the indicated time points were normalized to the baseline levels and represented as fold changes. (C) Correlation of fold changes of ICOS^high^PD‐1^high^ T_FH_ cells at day 37 with fold changes of SARS‐CoV‐2 neutralizing antibody at day 60 relative to those at baseline in patients with AR. (D and E) Frequencies of circulating ICOS^high^PD‐1^high^ T_FH_ cell subsets were analyzed by flow cytometry. Changes of ICOS^high^PD‐1^high^ T_FH_ cell subsets at the indicated time points were normalized to the baseline levels and represented as fold changes. (F) Correlations of fold changes of ICOS^high^PD‐1^high^ T_FH_ cell subsets at day 37 with fold changes of SARS‐CoV‐2 neutralizing antibody at day 60 in patients with AR. (G) Plasma SARS‐CoV‐2 neutralizing antibody and IgG levels in recovered COVID‐19 patients at 10–12 months after infection (*n* = 64 for controls without AR and *n* = 14 for those with AR). Numbers indicate the frequency of cells in the gated region. For (A), (B), (E) and (G), data are presented as median and interquartile range and analyzed by Mann–Whitney *U*‐test. For (C) and (F), data are analyzed by Spearman's rank correlation

As both T_FH_ activation and the production neutralizing antibody were strongly elicited after the second dose of vaccine, we focused on the memory response after the second dose in order to understand the relationship between T_FH_ function and protective antibody response. In patients with AR, the fold changes of ICOS^high^PD‐1^high^ cT_FH_ at day 37 positively correlated with the fold changes of IgG and IgM at day 60 but, intriguingly, not the fold changes of neutralizing antibody, which are more important in evaluating protective humoral immunity (Figure 2C; Figure S5C). Based on CXCR3 and CCR6 expression, cT_FH_ cells can be classified into three major subsets, referred as T_FH_1, T_FH_2 and T_FH_17 cells.^6^ T_FH_2 and T_FH_17 cells are reported to support chronic humoral immunity, such as autoantibody production in autoimmune diseases, whereas T_FH_1 cells can support antibody response in infection and vaccine with relatively short duration.^7^ With ICOS^high^PD‐1^high^ cT_FH_ cells, the T_FH_1 and T_FH_2, but not T_FH_17 subsets, were strongly induced by the vaccination (Figure 2D,E; Figure S6A). Importantly, we noted a marked increase in the frequencies of T_FH_2 cells among either ICOS^high^PD‐1^high^ cT_FH_ cells or total CD4^+^ T cells in AR patients compared with healthy subjects (Figure S6A). As expected, the fold changes of T_FH_2 cells to baseline were also elevated in AR patients at days 7 and 37 (Figure 2E). Furthermore, only T_FH_2 activation, not T_FH_1 and T_FH_17 activation, was found to be positively correlated with neutralizing antibodies in patients with AR (Figure 2F), despite positive correlations between T_FH_1 or T_FH_17 activation and IgG and IgM in AR patients (Figure S6B). Therefore, such results suggest that T_FH_2 subset might play a prominent role in promoting protective humoral response to two‐dose inactivated vaccination against SARS‐CoV‐2.

To verify the influence of AR on the humoral response to SARS‐CoV‐2, we additionally recruited 78 recovered COVID‐19 patients, including 64 without AR and 14 with AR, who showed no difference in demographic characteristics (Table S2). Similar to the humoral response elicited by inactivated SARS‐CoV‐2 vaccine, patients with pre‐existing AR displayed higher levels of neutralizing antibody 10–12 months after infection than control subjects, while the difference of SARS‐CoV‐2‐specific IgG did not reach statistical difference (Figure 2G).

Our study of AR patients for the antibody response to SARS‐CoV‐2 induced by inactivated vaccine or infection demonstrates that the protective humoral immune response was enhanced rather than compromised in AR patients. The prospective design in the vaccination study allowed us to measure both neutralizing antibody and RBD‐specific memory B cells, both supporting the above conclusion. As viral infection and vaccination, especially with influenza, induce a strong T_H_1 immune response, the generation and function of the T_FH_1 subset associated with T_H_1 response has been extensively investigated in viral infection and vaccination. Notably, COVID‐19 infection induces both T_H_1 and T_H_2 responses.^7^ Despite the T_FH_1 subset was variously activated by inactivated SARS‐CoV‐2 vaccine, our study revealed an unappreciated role of the T_FH_2 subset following vaccination. We have previously reported an increased T_FH_2 cells in patients with AR.^6^ Our new results demonstrate that the activation of T_FH_2 rather than T_FH_1 subset correlated with the induction of neutralizing antibodies in AR patients. Indeed, a recent study showed that IL‐4, a signature cytokine from T_FH_2 cells, plays a critical role in supporting broadly protective antibody response in viral infection.^9^ Although differing in intensity, similar humoral immune responses have been elicited by different SARS‐CoV‐2 vaccines.^10^ Whether AR patients have comparable immune responses to other SARS‐CoV‐2 vaccines, such as BNT162b2 mRNA vaccine, deserves further investigations.

In summary, our study provides the necessary evidence to justify the recommendation of SARS‐CoV‐2 vaccination to patients with AR and potentially other allergic diseases. Although the precise molecular mechanisms underlying the enhanced protective humoral immunity to COVID‐19 vaccine in patients with AR remain to be elucidated, the function of T_FH_2 cells in regulating anti‐SARS‐CoV‐2 humoral immune response should be a key area deserving a future investigation. In addition, it is also critical to understand whether type 2 response‐modifying treatments, such as allergen immunotherapy and biologics, will impair the efficacy of SARS‐CoV‐2 vaccination.

## CONFLICT OF INTEREST

The authors declare that there is no conflict of interest.

## Supporting information

Supporting Information

Figures S1–S6
