# Supplementary material for: TFH2 cells associate with enhanced humoral immunity to SARS‐CoV‐2 inactivated vaccine in patients with allergic rhinitis
Source: Clin Transl Med. 2022 Jan 26;12(1):e717. doi: 10.1002/ctm2.717 (PMC8792397; doi:10.1002/ctm2.717)
Supplement: Supplementary file 1 — Supporting Information [file CTM2-12-e717-s001.docx]

**Online Data Supplement**

**T_FH_2 cells associate with enhanced humoral immunity to SARS-CoV-2 inactivated vaccine in patients with allergic rhinitis**

Yin Yao, MD, PhD^1^*, Zhe-Zheng Wang, MD, PhD^1^*, Ao Huang, MD^1^*, Yan Liu, MD^1^, Nan Wang, MD, PhD^1^, Zhi-Chao Wang, MD, PhD^1^, Lin Yang, PhD^2^, Hui-Jun Li, MD^3^, Jun-Gang Xie, MD, PhD^4^, Rong-Fei Zhu, MD, PhD^2^, Li-Ming Cheng, MD, PhD^3^, Di Yu, PhD^5^, Zheng Liu, MD, PhD^1^

^1^Department of Otolaryngology-Head and Neck Surgery, Tongji Hospital, Tongji Medical College, Huazhong University of Science and Technology, Wuhan, China

^2^Department of Allergy, Tongji Hospital, Tongji Medical College, Huazhong University of Science and Technology, Wuhan, China

^3^Department of Laboratory Medicine, Tongji Hospital, Tongji Medical College, Huazhong University of Science and Technology, Wuhan, China

^4^Department of Respiratory and Critical Care Medicine, Tongji Hospital, Tongji Medical College, Huazhong University of Science and Technology, Wuhan, China

^5^The University of Queensland Diamantina Institute, Faculty of Medicine, The University of Queensland, Brisbane, Australia

*These authors contributed equally to this work.

**METHODS**

**Clinical study design and participants**

We first conducted a prospective cohort study to investigate the potential impact of AR on immune response and adverse events to SARS-CoV-2 vaccination. The trial is registered at https://clinicaltrials.gov (NCT05009134). Twenty-five healthy adults and 32 patients with AR were recruited at Tongji Hospital. The diagnosis of AR was based on the concordance between atopic status and typical allergic symptoms according to the Allergic Rhinitis and its Impact on Asthma (ARIA) guideline^S1^. Atopic status was evaluated by the skin prick test and/or ImmunoCAP (Phadia, Uppsala, Sweden) to detect specific IgE for common inhalant allergens in our region^S2^. AR patients had at least 1 year disease history and had never been infected with SARS-CoV-2 (no history of positive polymerase chain reaction results on nasopharyngeal or pharyngeal swab samples and levels of anti-SARS-CoV-2 spike and nucleocapsid protein IgG and IgM less than 10 AU/mL before vaccination). The exclusion criteria included: (i) the presence of sinusitis, (ii) pregnancy or breastfeeding, (iii) with severe immunologic diseases, diabetes, tumors, chronic kidney diseases, chronic obstructive pulmonary disease, cardiovascular diseases, stroke, or chronic infections, and (iv) used antihistamines or intranasal steroid in the past 1 week or oral steroids in the past 3 months prior to this study. Concomitant asthma and atopic eczema were diagnosed according to Global Initiative for Asthma guideline^S3^ and the Williams criteria^S4^, respectively. AR patients received intranasal antihistamine or intranasal steroid treatment as needed, and no systemic medication including oral steroids and antihistamines were allowed during this study. A visual analog scale (VAS) ranging from 0 (not at all bothersome) to 10 cm (extremely bothersome) was used to quantify the global perception of severity of combined nasal symptoms. Healthy controls had no history of allergy and had negative skin prick test and allergen specific IgE test.^S5^

All participants received an inactivated SARS-CoV-2 vaccine (WIBP-CorV, Sinopharm, Wuhan) on day 0 and day 30. Adverse events related to vaccination were recorded. Peripheral blood samples were collected at day 0 (baseline, before vaccination), day 7 (7 days after first vaccination), day 30 (30 days after first vaccination), day 37 (7 days after second vaccination), and day 60 (30 days after second vaccination). None were lost to follow-up. Plasma and peripheral blood mononuclear cells (PBMCs) were collected and stored as we previously described.^S6^

In addition, 78 recovered COVID-19 cases, who were infected with SARS-CoV-2 during January 13, 2020, to March 9, 2020, were retrospectively enrolled to validate the effect of AR on protective antibody response to SARS-CoV-2. Peripheral bloods were collected at 10 to 12 months after symptom onset. The diagnosis of COVID-19 was based on the guidance for diagnosis and management of COVID-19 released by World Health Organization and confirmed by SARS-CoV-2 polymerase chain reaction detection of nasopharyngeal or pharyngeal swab specimens at Tongji Hospital.^S7^ AR, asthma, and atopic eczema were determined based on patients' self-report on admission and further conformed by experienced physicians. Patients with bronchiectasis, cystic fibrosis, severe immunologic diseases, tumors, diabetes, chronic kidney diseases, chronic obstructive pulmonary disease, cardiovascular diseases, stroke, and sinusitis were excluded from the study. COVID-19 disease severity was classified as we previously reported.^S7^ The exclusion criteria led to only subjects with mild and moderate COVID-19 included in this retrospective study.

This study was approved by the Tongji Hospital Ethics Committee. Informed consent was obtained from every participant. All blood samples were processed and analyzed by investigators blinded to clinical data of patients. All samples were used in accordance with approved guidelines and regulations.

**Adverse events**

For the first 7 days after each dose, participants involved in vaccine study were provided with a daily record card for recording the injection site symptoms (pain, redness, and swelling), systematic symptoms (fatigue, fever, headache, and myalgia), digestive symptoms (diarrhea, vomiting, and nausea), respiratory symptoms (runny nose, cough, stuffy nose, and sore throat), and allergic symptoms (pruritus, hives, itchy throat, swelling of mouth o throat, shortness of breath, and anaphylaxis).^S8^ From day 8 to day 28 after each dose, safety data were collected by spontaneous report from the participants combined with the regular visit. Adverse events were further verified by investigators and the causal association between adverse events and vaccination was determined by the investigators.

**Plasma antibodies detection**

The neutralizing antibody against the receptor-binding domain (RBD) of the SARS-CoV-2 spike 1 protein in plasma were measured by chemiluminescent immunoassay using iFlash 2019-nCoV neutralization assay kits (YHLO Biotech Co, Shenzen, China) according to the manufacture’s instruction. This surrogate neutralization assay detects antibodies that are able to compete with binding of a recombinant extracellular domain of the SARS-CoV-2 receptor angiotensin-converting enzyme 2 to a recombinant RBD of the SARS-CoV-2 spike 1 protein coated on microparticles and thus indicates the neutralization activity of sera.^S9^ The SARS-CoV-2 neutralizing antibody concentrations were measured by an iFlash3000 Chemiluminescence Immunoassay Analyzer (YHLO Biotech Co). Plasma IgM and IgG specific to the spike and the nucleocapsid proteins of the SARS-CoV-2 were detected using chemiluminescent immunoassay kits (Yhlo Biotech Co.) as we previously reported.^S10^

**Flow cytometry**

After blocking with 5% fetal bovine serum (FBS, Thermo Fisher Scientific, Waltham, MA, USA), PBMCs were stained with Zombie Aqua (Biolegend, San Diego, CA, USA) to exclude dead cells. Then, cells were incubated with fluorochrome-labeled antibodies for 30 min at 25°C to label surface marker. Isotype controls were used as negative controls. Fluorescence minus one control for each fluorochrome was used to set the gate for positive populations. Data were analyzed using a BD LSRFortessa™ X-20 (BD Bioscience) instrument and FlowJo software (TreeStar, Ashland, OR, USA) as previously described.^S6^ Details of antibodies used flow cytometry are tabulated in Table S3.

To detect SARS-CoV-2-specific B cells, biotinylated recombinant SARS-CoV-2 Spike RBD protein antigens (BT10500, R&D systems) were individually multimerized with fluorescently labeled streptavidin at 4°C for 1 hour as previously described.^S11,12^ Briefly, recombinant SARS-CoV-2 RBD protein was mixed with AF647-streptavidin (Biolegend) or Perp5.5-streptavidin (Biolegend) at 2:1 ratio (~4:1 molar ratio) at 4°C for 1 hour. The antigen probes prepared individually as above were then mixed in 50 mM free d-biotin (Macklin) in PBS to reduce cross-reactivity. PE-streptavidin (BioLegend) was used as a decoy probe to gate out SARS-CoV-2 non-specific streptavidin-binding B cells. PBMC (5×10^6^) were prepared in U-bottom 96-well plates and stained with 50 μL of antigen probe cocktail containing 50 ng of RBD and 20 ng of streptavidin-PE at 4°C for 30 min. After washing, cells were then stained with 50 μL of antigen probe cocktail containing.

**Statistical analysis**

All statistical analyses were carried out with GraphPad Prism Software v.9.0 (GraphPad Software). Data are presented in dot plots by medians and interquartile ranges unless specifically stated. Data were analyzed by the Mann–Whitney *U*-test or paired-sample *t*-test. For categorical data, chi-squared test or Fisher’s exact test was performed. Spearman’s rank correlation analysis was used to analyze associations. A *P* value < 0.05 was considered statistically significant.

**REFERENCES:**

S1. Bousquet J, Khaltaev N, Cruz AA, et al. Allergic Rhinitis and its Impact on Asthma (ARIA) 2008 update (in collaboration with the World Health Organization, GA(2)LEN and AllerGen). Allergy 2008;63(suppl 86):8-160.

S2. Zhu R, Wang J, Wu Y, et al. Allergic Rhinitis Control Test questionnaire-driven stepwise strategy to improve allergic rhinitis control: a prospective study. Allergy 2016;71:1612-1619.

S3. Bateman ED, Hurd SS, Barnes PJ, et al. Global strategy for asthma management and prevention: GINA executive summary. Eur Respir J 2008;31:143-78.

S4. Williams HC, Burney PG, Hay RJ, et al. The U. K. Working Party’s Diagnostic Criteria for Atopic Dermatitis. I. Derivation of a minimum set of discriminators for atopic dermatitis. Br J Dermatol 1994;131:383-396.

S5. Yao Y, Wang ZC, Wang N, et al. Allergen immunotherapy improves defective

follicular regulatory T cells in patients with allergic rhinitis. J Allergy Clin Immunol

2019;144:118-128

S6. Yao Y, Chen Z, Zhang H, et al. Selenium-GPX4 axis protects follicular helper T

cells from ferroptosis. Nat Immunol 2021;22:1127-1139.

S7. Song J, Zeng M, Wang H, et al. Distinct effects of asthma and COPD comorbidity

on disease expression and outcome in patients with COVID-19. Allergy 2021;

2021;76:483-496.

S8. Jara A, Undurraga EA, Gonzalez C, et al. Effectiveness of an Inactivated

SARS-CoV-2 Vaccine in Chile. N Engl J Med 2021;385:875-884.

S9. Tenbusch M, Schumacher S, Vogel E, et al. Heterologous prime-boost vaccination

with ChAdOx1 nCoV-19 and BNT162b2. Lancet Infect Dis 2021;21:1212-1213.

S10. Liu L, Chen HG, Li Y, et al. Temporal profiles of antibody responses,

cytokines, and survival of COVID-19 patients: A retrospective cohort in Wuhan,

China. Engineering (Beijing) 2021;7:958-965.

S11. Dan JM, Mateus J, Kato Y, et al. Immunological memory to SARS-CoV-2

assessed for up to 8 months after infection. Science 2021;371:eabf4063.

S12. Zhang H, Liu Y, Liu D, et al. Time of day influences immune response to an

inactivated vaccine against SARS-CoV-2. Cell Research 2021; 31:1215-1217.

**Figure Legends**

**Figure S1 Changes of** **SARS-CoV-2 neutralizing antibody, IgG, and IgM levels after inactivated SARS-CoV-2 vaccination.** Healthy subjects (*n* = 25) and patients with AR (*n* = 32) were enrolled and received inactivated SARS-CoV-2 vaccine at day 0 and day 30. Peripheral blood was collected at day 0 (baseline), day 7, day 30, day 37, and day 60. **a,** Fold changes of SARS-CoV-2 neutralizing antibody, IgG, and IgM levels normalized to the baseline levels (day 0). Data are analyzed by two-sided paired-sample *t*-test. **b,** Correlation of the levels of SARS-CoV-2 neutralizing antibody and IgG. Data are analyzed by Spearman’s rank correlation. **c,** SARS-CoV-2 neutralizing antibody, IgG, and IgM levels in healthy controls and patients with AR. Data are presented as median and interquartile range and analyzed by Mann–Whitney *U*-test.

**Figure S2 Flow cytometric gating strategy of B cell subsets in human peripheral bloods.** Within viable CD3^–^CD19^+^CD20^+^ cells, naive B cells were defined as CD27^-^IgD^+^, nonswitched memory B cells as CD27^+^IgD^+^, switched memory B cells as CD27^+^IgD^-^, and double negative B cells as CD27^-^IgD^-^. Within viable CD3^–^CD19^+^ cells, plasmablasts were defined as CD27^+^CD38^++^.

**Figure S3 Circulating B cell subsets at day 60 after inactivated SARS-CoV-2 vaccination.** Frequencies of circulating B cell subsets in healthy controls (*n* = 25) and AR patients (*n* = 32) were analysed by flow cytometry. Data are presented as median and interquartile range and analyzed by Mann–Whitney *U*-test.

**Figure S4 Flow cytometric gating strategy of T cell subsets in human peripheral bloods.** Within viable CD3^+^CD8^–^CD4^+^ T cells, T_REG_ cells were defined as CD25^high^CD127^low^ cells, T_FR_ cells as CD45RA^–^CXCR5^+^ T_REG_ cells, circulating T_FH_ cells as CD25^–/low^CD45RA^–^CXCR5^+^ICOS^high^PD-1^high^ cells, T_FH_1 cells as CXCR3^+^CCR6^–^ T_FH_ cells, T_FH_2 cells as CXCR3^–^CCR6^–^ T_FH_ cells, and T_H_17 cells as CXCR3^–^CCR6^+^ T_FH_ cells.

**Figure S5 Changes of circulating T cell subsets after inactivated SARS-CoV-2 vaccination.** **a**, Frequencies of circulating T cell subsets in healthy controls (*n* = 25) and patients with AR (*n* = 32) were analysed by flow cytometry. **b**, Changes of frequencies of T cell subsets at the indicated time points were normalised to the baseline levels and represented as fold changes. Data are presented as median and interquartile range and analyzed by Mann–Whitney *U*-test. **c**, Correlations of fold changes of T_FH_ cells at day 37 relative to those at day 0 with changes of levels of SARS-CoV-2 IgG and IgM at day 60 relative to those at day 0 in patients with AR. Data are analyzed by Spearman’s rank correlation.

**Figure S6 Changes of circulating T_FH_ cell subsets after inactivated SARS-CoV-2 vaccination.** **a**, Frequencies of circulating ICOS^high^PD-1^high^ T_FH_ cell subsets in healthy controls (*n* = 25) and AR patients (*n* = 32) were analysed by flow cytometry. Data are presented as median and interquartile range and analyzed by Mann–Whitney *U*-test. **b**, Correlations of fold changes of ICOS^high^PD-1^high^ T_FH_ cell subsets at day 37 relative to those at day 0 with changes of levels of SARS-CoV-2 IgG and IgM at day 60 relative to those at day 0 in patients with AR. Data are analyzed by Spearman’s rank correlation.

**Table S1 Adverse events related to COVID-19 vaccination**

|  | **First dose** | | | **Second dose** | | |
| --- | --- | --- | --- | --- | --- | --- |
|  | **Healthy subjects**  **(*n* = 25)** | **AR patients**  **(*n* = 32)** | ***p* value** | **Healthy subjects**  **(*n* = 25)** | **AR patients**  **(*n* = 32)** | ***p* value** |
| Participants with adverse events, n (%) | 10 (40%) | 13 (43.8%) | 0.962 | 9 (36%) | 11 (34.4%) | 0.899 |
| **Injection site symptom**  Pain, N (%)  Redness, N (%),  Swelling, N (%) | 9 (36%)  1 (4%)  1 (4%) | 10 (31.2%)  4 (12.5%)  2 (6.3%) | 0.706  0.513  0.826 | 8 (32%)  1 (4%)  0 | 7 (21.9%)  3 (9.4%)  2 (6.3%) | 0.389  0.790  0.499 |
| **Systematic symptom**  Fever, N (%)  Fatigue, N (%)  Headache, N (%)  Myalgia, N (%) | 0  4 (16%)  1 (4%)  3 (12%) | 2 (6.3%)  8 (25%)  1 (3.1%)  4 (12.5%) | 0.499  0.408  1.000  0.727 | 0  1 (4%)  0  1 (4%) | 0  4 (12.5%)  1 (3.1%)  1 (3.1%) | -  0.513  1.000  1.000 |
| **Digestive symptom**  Diarrhea, N (%)  Vomiting, N (%)  Nausea, N (%) | 0  0  0 | 0  0  0 | -  -  - | 0  0  0 | 0  0  1 (3.1%) | -  -  1.000 |
| **Respiratory symptom**  Runny nose, N (%)  Cough, N (%)  Stuffy nose, N (%)  Sore throat, N (%) | 0  0  0  1 (4%) | 1 (3.1%)  0  1 (3.1%)  2 (6.3%) | 1.000  -  1.000  0.826 | 0  0  0  0 | 1 (3.1%)  0  1 (3.1%)  0 | 1.000  -  1.000  - |
| **Allergic symptom**  Pruritus, N (%)  Hives, N (%)  Itchy throat, N (%)  Swelling of mouth or throat, N (%)  Shortness of breath, N (%)  Anaphylaxis, N (%) | 1 (4%)  0  0  0  0  0 | 1 (3.1%)  0  0  0  0  0 | 1.000  -  -  -  -  - | 0  0  0  0  0  0 | 1 (3.1%)  0  0  0  0  0 | 1.000  -  -  -  -  - |

AR, allergic rhinitis

**Table S2 Demographic characteristics of recovered COVID-19 cases**

|  | **Without AR** | **With AR** | ***p* value** |
| --- | --- | --- | --- |
| Total subjects, n | 64 | 14 | - |
| Gender, male/female | 10/54 | 2/12 | 0.777 |
| Age (years) | 31 (27, 36) | 29 (27, 32) | 0.849 |
| Patients with asthma, n (%) | 0 | 1 (7.1%) | 0.180 |
| Patients with atopic eczema, n (%) | 0 | 1 (7.1%) | 0.180 |
| Disease severity  Mild, n (%)  Moderate, n (%) | 18 (28.1%)  46 (71.9%) | 3 (21.4%)  11 (78.5%) | 0.858  0.858 |
| Sampling time after symptom onset (months) | 11 (11, 12) | 11 (11, 12) | 0.635 |

For continuous variables, data are expressed as medians and interquartile ranges. AR, allergic rhinitis

**Table S3 Antibodies used for flow cytometry**

| **Antibody** | **Parameter** | **Clone ID** | **Source** | **Isotype** | **Manufacturer** | **Dilution** |
| --- | --- | --- | --- | --- | --- | --- |
| CCR6 | PE | G034E3 | mouse | IgG2b, κ | Biolegend  (San Diego,  CA, USA) | 1:100 |
| CCR7 | Alexa Fluor® 647 | G043H7 | mouse | IgG2a κ | Biolegend | 1:100 |
| CXCR3 | PE-Cy7 | G025H7 | mouse | IgG1, κ | Biolegend | 1:100 |
| CXCR5 | BB515 (FITC) | RF8B2 | Rat | IgG2b, κ | BD Biosciences (San Jose, CA, USA) | 1:100 |
| CD127 | Brilliant Violet 711 | A019D5 | mouse | IgG1, κ | Biolegend | 1:100 |
| CD19 | Alexa Fluor® 700 | HIB19 | mouse | IgG1, κ | Biolegend | 1:200 |
| CD20 | Brilliant Violet 785 | 2H7 | mouse | IgG2b, κ | Biolegend | 1:200 |
| CD25 | APC-Cy7 | BC96 | mouse | IgG1, κ | Biolegend | 1:50 |
| CD27 | PE-Cy7 | M-T271 | mouse | IgG1, κ | Biolegend | 1:100 |
| CD3 | Brilliant Violet 711 | OKT3 | mouse | IgG2a, κ | Biolegend | 1:200 |
| CD38 | BB515 (FITC) | HIT2 | mouse | IgG1, κ | Biolegend | 1:200 |
| CD4 | Brilliant Violet 605 | RPA-T4 | mouse | IgG1, κ | Biolegend | 1:200 |
| CD45RA | PerCP-Cy5.5 | HI100 | mouse | IgG2b, κ | Biolegend | 1:200 |
| CD8 | Alexa Fluor® 700 | RPA-T8 | mouse | IgG1, κ | Biolegend | 1:200 |
| ICOS | Brilliant Violet 785 | C398.4A | Arrnenian Hamster | IgG | Biolegend | 1:100 |
| IgD | APC-Cy7 | IA6-2 | mouse | IgG2a, κ | BD Biosciences | 1:200 |
| IgG | Brilliant Violet 421 | M1310G05 | Rat | IgG2a, κ | Biolegend | IgG1, κ |
| IgM | Brilliant Violet 605 | MHM-88 | Mouse | IgG1, κ | IgG1, κ | IgG1, κ |
| PD-1 | Brilliant Violet 421 | EH12.2H7 | mouse | IgG1, κ | IgG1, κ | 1:100 |

PE, phycoerythrin; APC, allophycocyanin; PerCP, peridinin chlorophyll protein complex; FITC, fluorescein isothiocyanate.
