# Supplementary figures and images for: TFH2 cells associate with enhanced humoral immunity to SARS‐CoV‐2 inactivated vaccine in patients with allergic rhinitis
Source: Clin Transl Med. 2022 Jan 26;12(1):e717. doi: 10.1002/ctm2.717 (PMC8792397; doi:10.1002/ctm2.717)

Figure S1

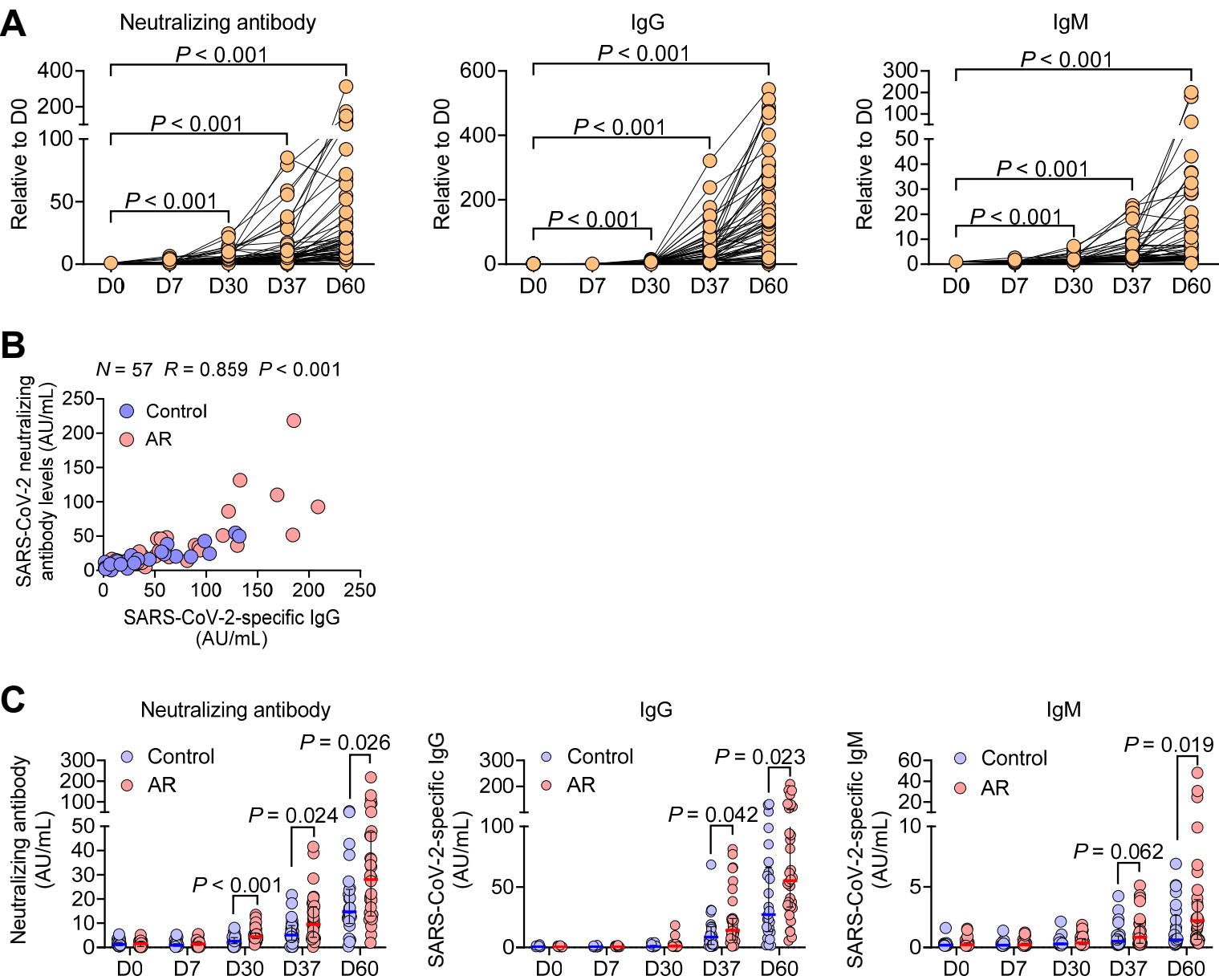

**Figure S2**

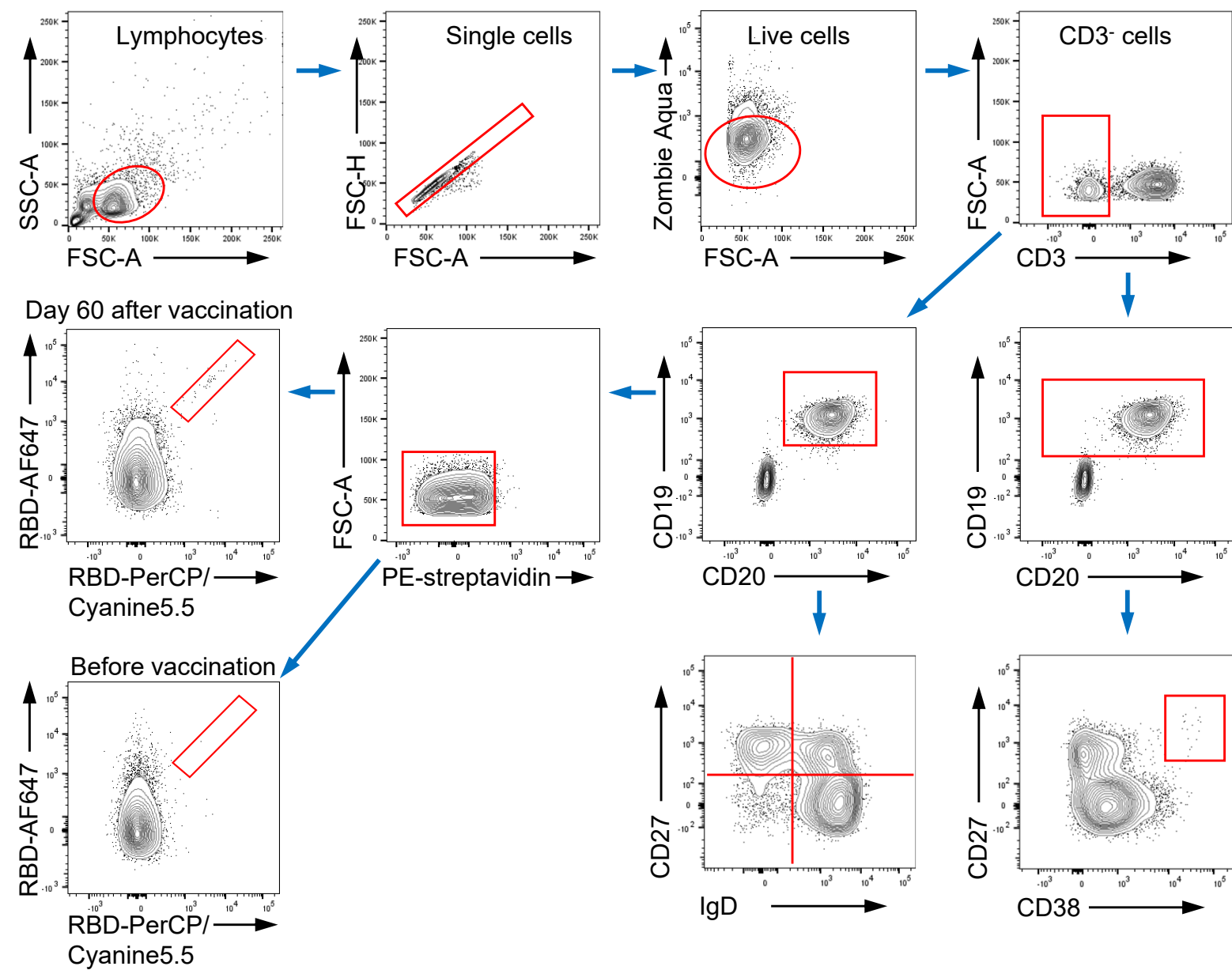

Figure S3

A

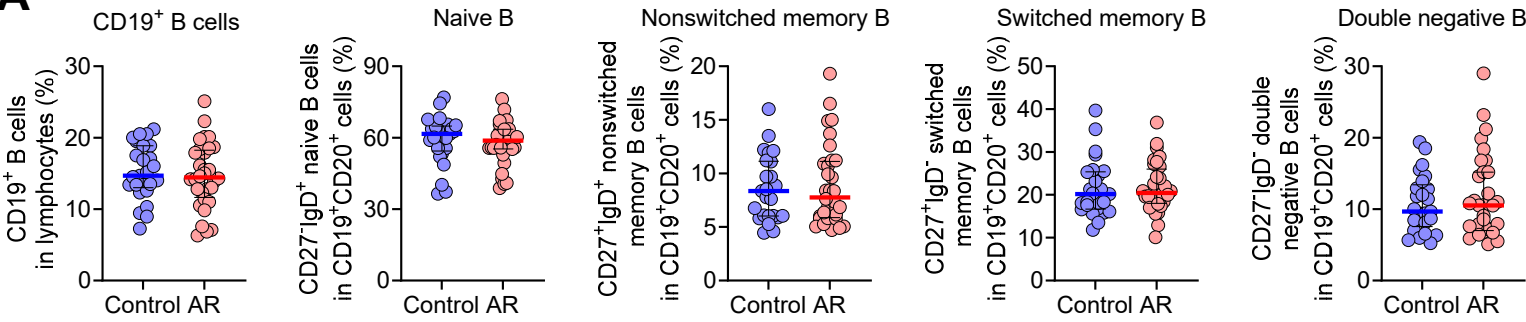

B

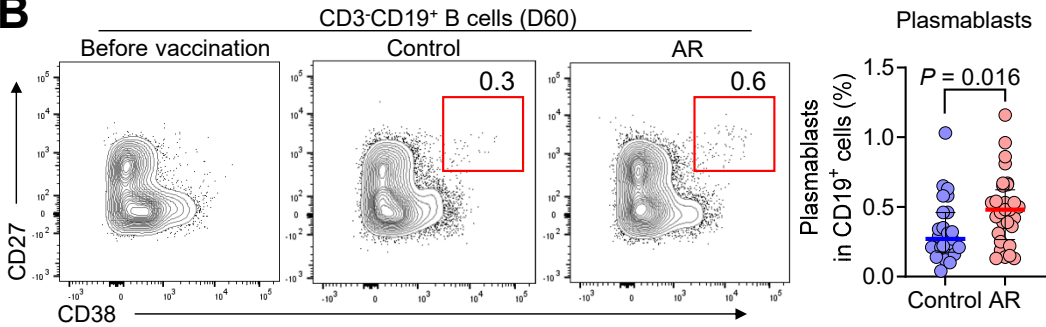

**Figure S4**

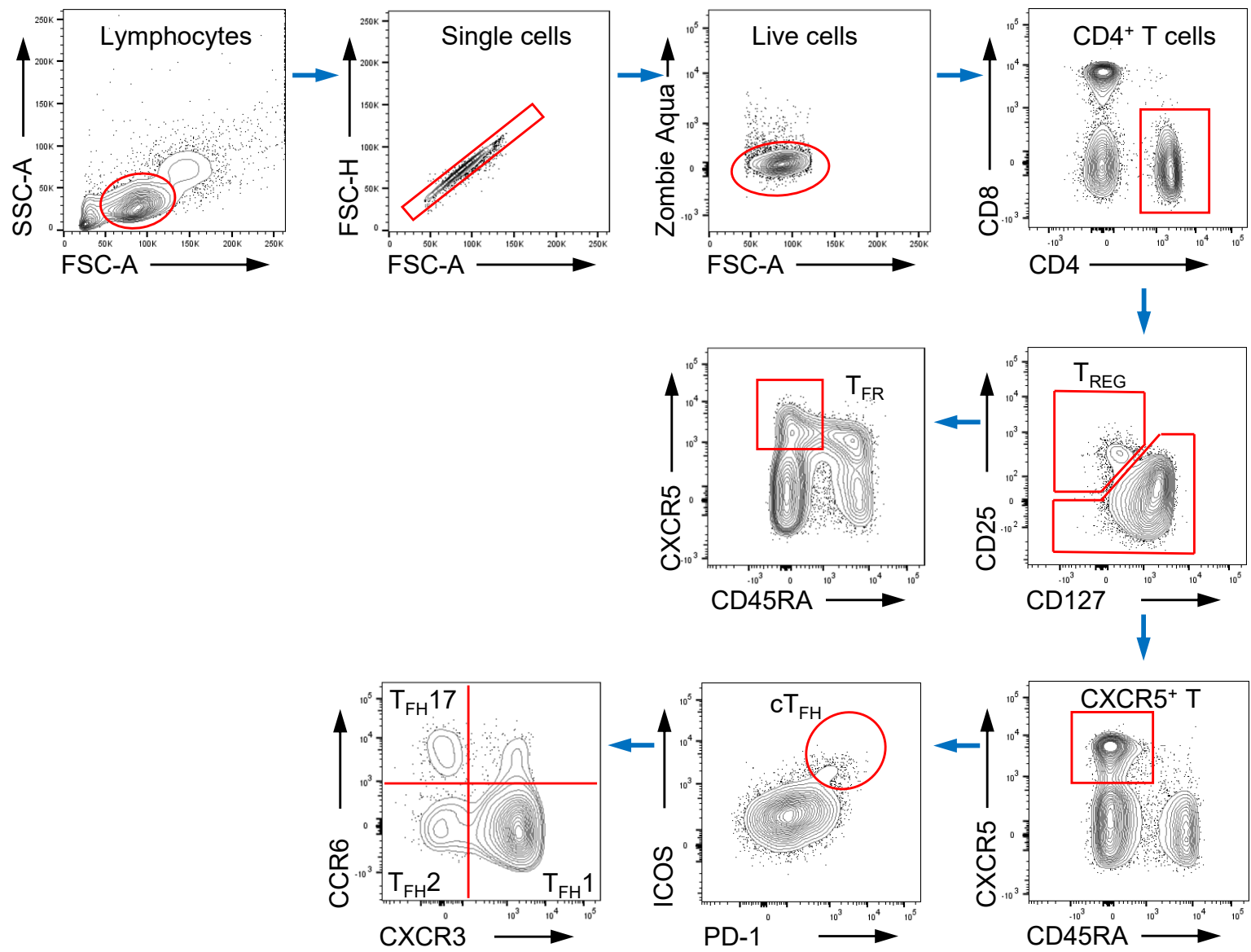

Figure S5

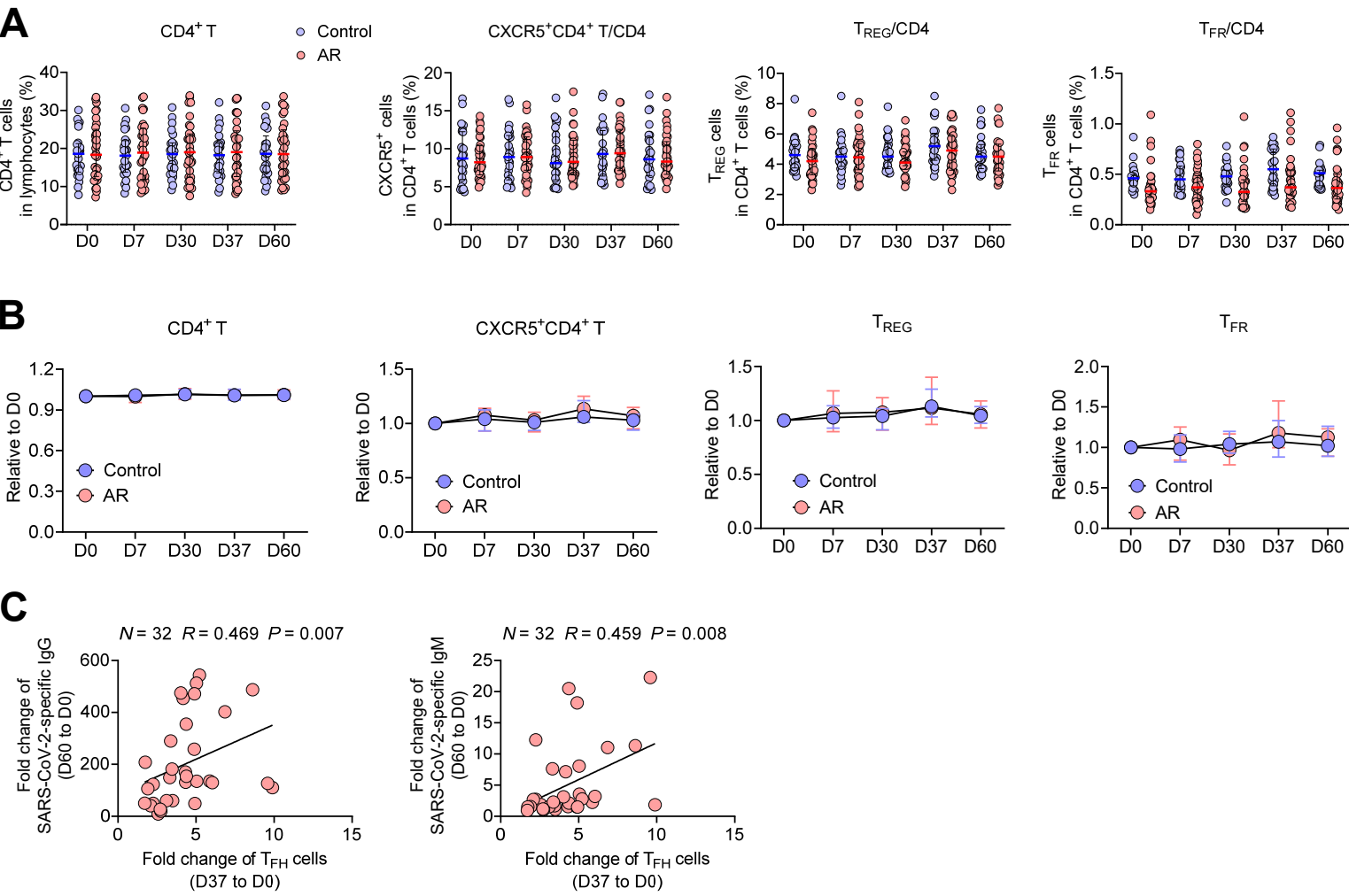

# Figure S6

**A**

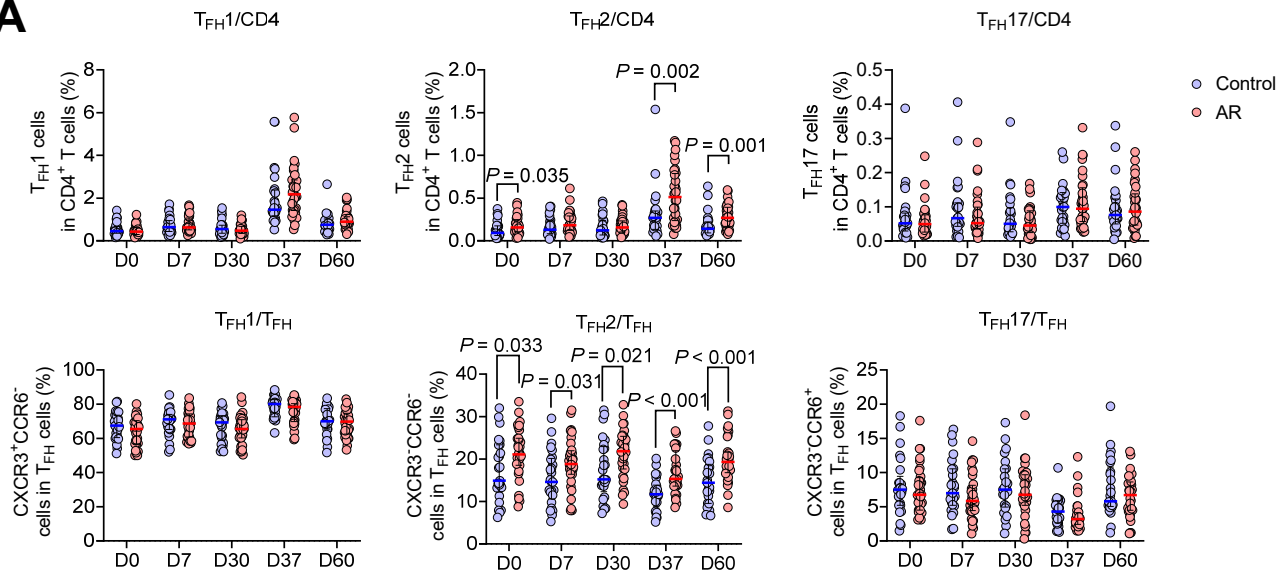

**B**

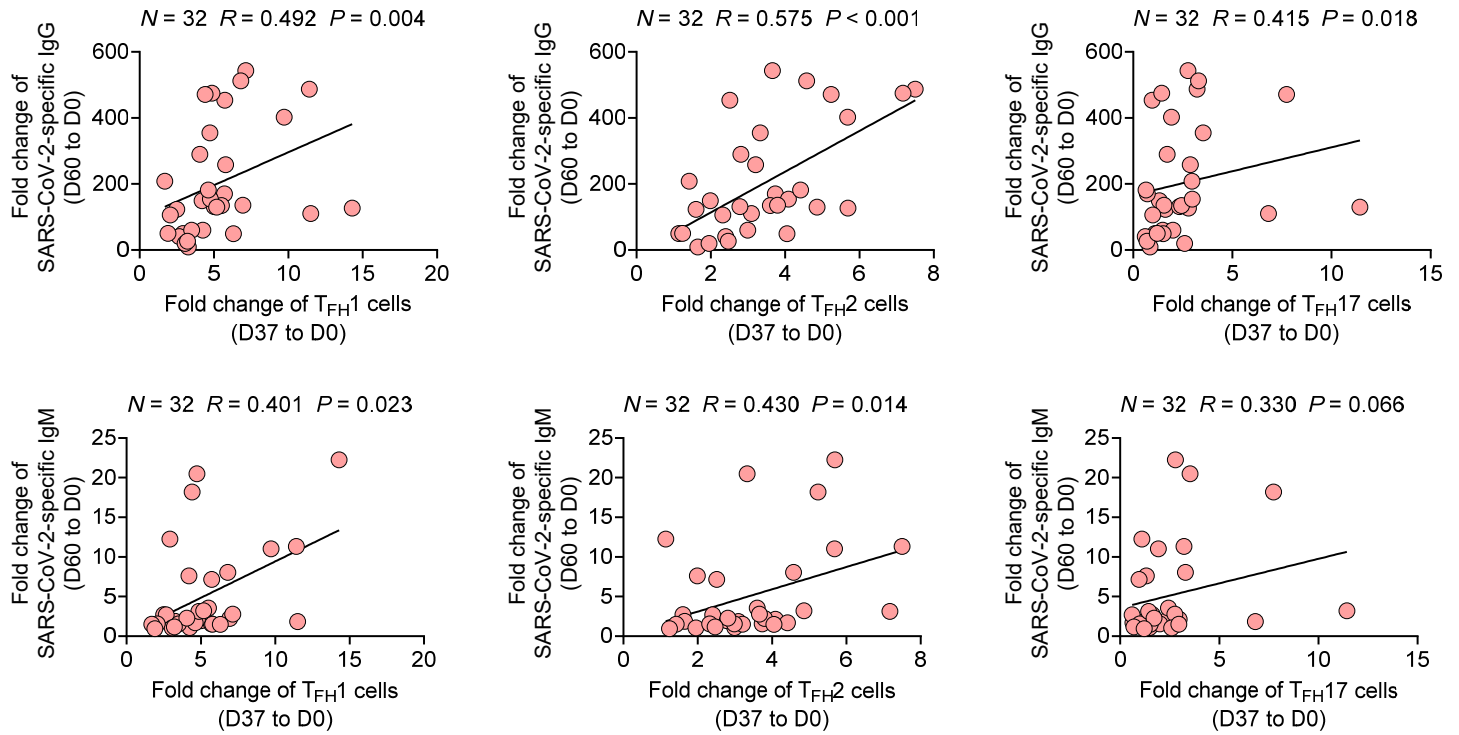

Supplement: Supplementary file 2 — Figures S1–S6 [file CTM2-12-e717-s002.pdf]
